# Visualization and Analysis of Global Vision Zero Studies and Policy Orientation in China

**DOI:** 10.3390/ijerph192214841

**Published:** 2022-11-11

**Authors:** Yi He, Yixiong Fan, Lixin Yan, Jianhua Peng, Zhiqiang Li

**Affiliations:** 1 Intelligent Transportation Research Center, Wuhan University of Technology, Wuhan 430063, China; 2 National Engineering Research Center for Water Transport Safety, Ministry of Science and Technology of the People’s Republic of China, Wuhan 430063, China; 3 School of Transportation Engineering, East China Jiaotong University, Nanchang 330013, China; 4 China Academy of Transportation Science, Ministry of Transport of the People’s Republic of China, Beijing 100029, China

**Keywords:** Vision Zero, road safety, visualization analysis, mapping knowledge domain, VOSviewer

## Abstract

As a policy that is widely used worldwide, Vision Zero is a long-term strategic goal for road safety in China. The aim of this paper is to examine the literature in the field of Vision Zero retrieved by the Web of Science (WOS) Core Collection database from 1997 to 2021 based on mapping knowledge domain (MKD) and bibliometric methods. In this paper, we analyzed the overall development level of Vision Zero at different stages using the statistical analysis of the distribution of literature years. Based on the analysis, four major research directions of Vision Zero are obtained through keywords co-occurrence analysis, including “Vision Zero in road safety”, “vulnerable road users’ risks”, “effect of speed on severity”, and “responsibility management for crashes”. Furthermore, we explore the influence and development potential of each country and region of Vision Zero based on the number and citation frequency of the literature, and the distribution of time dimensions. Among the research topics, all countries and regions are clustered into four clusters, and the current status of Vision Zero for countries in the most influential clusters, which include Sweden, the United States, Australia, China, and Norway, have been presented. Finally, an analysis of road safety in China is carried out, which includes displaying the changes in the number of road accidents, fatalities, and serious injuries in China between 2009 and 2018, comparing the fatalities per 10,000 vehicles and the fatalities per 100,000 people between China and other countries, and describing China’s strategic routes toward Vision Zero.

## 1. Introduction

With the development of the transportation industry, the global level of motorization continues to improve, whereas the cost of this rapid growth motorization is high. According to the data released by the World Health Organization, about 1.35 million people are killed by road accidents every year, while between 20 million and 50 million people suffer non-fatal injuries, many of them resulting in disability [1]. Although reducing the number of road accidents and fatalities has become a global priority, bureaucratic safety systems have resulted in few countries taking drastic action to achieve it [2,3]. Having innovative and radical political decisions is essential to address road safety issues.

The Swedish Parliament passed a road safety bill named Vision Zero in 1997, which means that Vision Zero became the basis of road safety in Sweden and that the Swedish road transport system will be adapted to human psychological and physical conditions and limitations [4]. Vision Zero introduces a new philosophy to global road safety: people should not be at risk of dying while using the road system [5]. Any accident with serious consequences is socially unacceptable and needs to be prevented by every means possible [6,7,8,9]. This vision has some similarities to today’s safety policies in industries, such as mining, nuclear power plants, aviation, and rail operations [10]. Prior to Vision Zero, most people did not consider maximizing human life as a fundamental principle that outweighed all other goals [11].

With the rising awareness of people’s safety, road accidents have become a major public health issue that has drawn the key attention of the government and the public [12]. In recent years, many countries have started to formulate their own Vision Zero acts and are eager to establish a people-oriented and better, safer transportation system. It is meaningful to identify the coverage and gaps in Vision Zero through a large number of research papers to obtain a comprehensive understanding of the current status of road safety in countries around the world [13]. Most previous studies have discussed and summarized a limited number of research papers based on a subjective descriptive approach that does not adequately reflect the intrinsic relevance of that research area. In this context, it is a challenging study to conduct a comprehensive analysis of papers in the field of Vision Zero.

As the most populous country in the world, China has the second largest number of road fatalities, and there is a considerable gap between the fatalities per 10,000 vehicles and that of the developed countries. Chinese Ministry of Transport propose to basically build China’s strength in transportation by 2035 and become the leading power in the world in terms of transportation by 2050. Traffic safety is the premise and guarantee of building a transportation power, and Vision Zero is the continuous pursuit of traffic safety [14]. Vision Zero is highly valued in China, but there is a lack of relevant research. Thus, China is selected as the focal point of Vision Zero in this paper.

Mapping knowledge domain is a method for describing newly developed interdisciplinary scientific fields, aiming to simplify access the information and clarify knowledge structures [15]. It can also be used to study the current state of international research and hot frontiers of knowledge dissemination. Bibliometric analysis and mapping knowledge domain are widely used in the field of the literature review and analysis due to their advantages of intuitive quantitative statistics, clear visual graphical display, objective description, and evaluation [16].

This paper explores the current state of Vision Zero and visualizes it based on mapping knowledge domain and bibliometric methods, provides an objective summary of the road safety situation in China, and offers ideas for future research directions.

## 2. Methodology

This research takes the literature system and the literature characteristics as the research object and uses bibliometric analysis to obtain the quantity, distribution relationship, and clustering results of the literature. The complex knowledge domain is visualized through the mapping knowledge domain, and the dynamic development pattern of Vision Zero is clearly and explicitly revealed.

### 2.1. Data Source and Search Content

This article uses the Web of Science (WOS) Core Collection database as the source of the literature data, with the search keyword “Vision Zero”, and the search time span is from 2002 to 2021. In the end, a total of 142 published related papers are collected, and the last one was updated in May 2021. All collected papers are written in English.

### 2.2. Analysis Tool and Methods

The analysis tool used in this study is VOSviewer, which specializes in mapping knowledge domains and clustering of documents [17]. In this paper, VOSviewer visualizes and analyzes the literature in the field of Vision Zero based on similarity visualization technique and uses the co-occurrence matrix for a layout to generate a knowledge graph. The core method consists of two parts: constructing the similarity matrix and VOS layout.

#### 2.2.1. Construction of Co-Occurrence Matrix

The similarity matrix is obtained by normalizing the co-occurrence matrix and correcting the differences in the total occurrence times or common frequency of elements in the co-occurrence matrix [18]. The similarity $S_{ij}$ between two items i and j is calculated as:

(1) $$\begin{array}{c} S_{ij}=\frac{C_{ij}}{W_{i}W_{j}}\;\;\; \end{array}$$

where $S_{ij}$ is the similarity between element i and element j, $C_{ij}$ is the number of co-occurrences of element i and element j, and $W_{i}$ and $W_{j}$ are the total number of occurrences of element i and element j, respectively.

#### 2.2.2. VOS Layout

The layout method of VOS uses the spatial distance between element i and element j to reflect the similarity $S_{ij}$, and the closer the elements are, the higher the similarity is indicated. Minimizing the sum of weighted Euclidean distances between all elements in each cluster can effectively improve the clustering effect. The distance of each cluster can be expressed by Equation (2).

(2) $$\begin{array}{c} E\left(x_{1},x_{2},\ldots ,x_{n}\right)=\underset{i<j}{\sum }S_{ij}\left\|X_{i}-X_{j}\right\|{}^{2} \end{array}$$

where n is the number of elements and ‖·‖ is the Euclidean parametrization. The minimization of the objective function should be constrained by Equation (3).

(3) $$\begin{array}{c} \;\frac{2}{n\left(n-1\right)}\underset{i<j}{\sum }\left\|X_{i}-X_{j}\right\|{}^{2}=1 \end{array}$$

## 3. Documents Statistical Analysis

### 3.1. Yearly Distribution of the Literature

**Figure 1:**
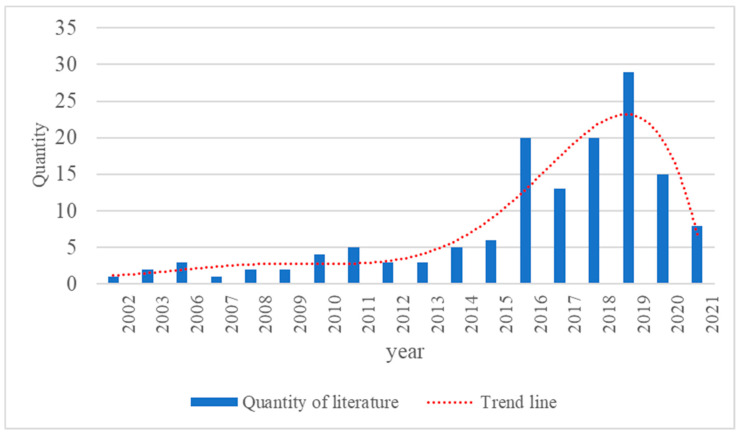
Quantitative distribution of the published literature in Vision Zero, 2002–2021.

The development of a subject area can be reflected by the number of documents in that field. The change in the number of documents reflects the attention paid to the field by researchers. Studying the distribution pattern of the literature over time can provide an understanding of the current level of development and future trends of the subject area. Figure 1 shows the statistics of the literature distribution in the field of Vision Zero by year.

As shown in Figure 1, there are 142 relevant documents in Vision Zero between 2002 and 2021. The developmental experience of these documents can be divided into two stages.

- First stage (2002–2015): From 2002 to 2015, there were few documents in this field, within five articles per year. Most of the literature in this stage is an exploration of the interpretation of Vision Zero and a discussion of some controversial points.
- Second stage (2016–2021): From 2016 to 2021, the related literature shows a rapid growth, which marks the beginning of the focus on Vision Zero as a research hotspot. The literature in this stage has focused on the application of Vision Zero to various transportation concepts.

### 3.2. Keywords Co-Occurrence Analysis

**Figure 2:**
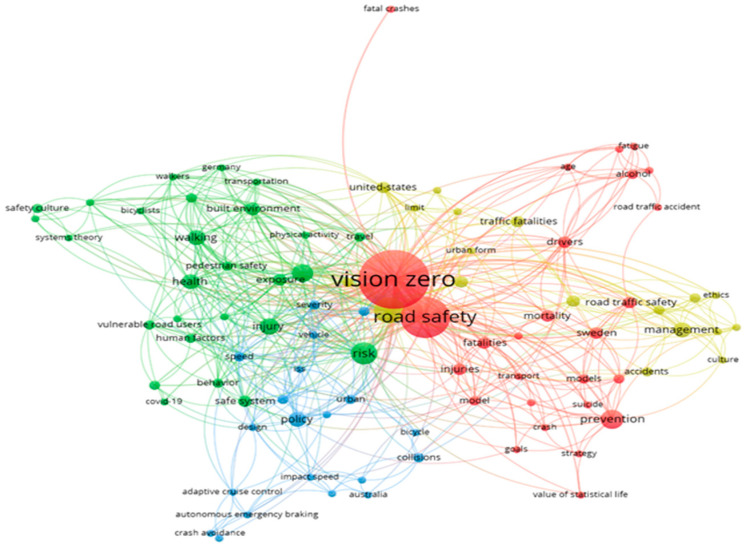
Keywords co-occurrence network of Vision Zero.

Keywords co-occurrence analysis is a method that uses high-frequency words to describe current research hotspots in the field. The principle is to perform relevance statistics based on the number of common occurrences of keywords between the different literature in a certain field, so as to obtain the knowledge structure relationship in the field. It is a commonly used scientific scientometric method. The use of keywords co-occurrence analysis allows the identification of important keywords used in Vision Zero and helps provide insights into the major research themes in the area. The Vision Zero keywords co-occurrence network diagram is shown in Figure 2.

As observed in Figure 2, keywords are clustered into a total of four clusters. The keywords clustered into the same cluster are highly related and represent a research topic. According to the keywords in the clusters, we can analyze and summarize the characteristics of each cluster.

(1) Cluster 1 (Red): Vision Zero in road safety

Keywords in cluster 1 (Red) include: Vision Zero, road safety, prevention, Sweden, fatalities, mortality, injuries, road traffic safety, drivers, alcohol, drugs, etc.

Vision Zero is a new policy focusing on road safety adopted by the Swedish government. Unlike traditional road safety policies, the concept of Vision Zero is to shift from accident prevention to the prevention of fatalities and serious injuries [19]. In other words, the ultimate goal of Vision Zero is to achieve a road system that is free of fatalities and serious injuries [20]. In recent years, the number of road fatalities has gradually moved in a positive direction, proving the effectiveness of the policy. Vision Zero’s success in road traffic safety has led to its adoption in several areas [21]. Although the research on Vision Zero is relatively limited [22], the concept has been used in numerous safety areas, such as aviation, rail, fire, drug use, workplace, etc. [10,23]. In fact, Vision Zero is easy to state, but turning Vision Zero into a viable tool in every field is difficult [23]. According to the World Health Organization, alcohol causes about 5% to 35% of all traffic fatalities [1]. Therefore, it is imperative to study the influences of alcohol on drivers. Current research on alcohol in China has mainly focused on the effects of alcohol on personal characteristics and driving behavior [24,25]. Despite the fact that many drivers drive after using drugs on a daily basis, quantitative studies of the influences of drug use on drivers are lacking due to the insidious nature of drugs and the complexity of detection.

(2) Cluster 2 (Green): Vulnerable road users’ risks

Keywords in cluster 2 (Green) include: risk, injury, walking, health, exposure, vulnerable road users, pedestrian safety, human factors, built environment, behavior, etc.

More than 90% of road accidents are caused by road users [26]. Most previous road safety policies have mostly targeted motorists while neglecting pedestrian safety. After the implementation of Vision Zero, there is still a lack of research on injuries to vulnerable road users [27]. A study shows that while serious injuries among car occupants have declined in recent years, the risk of serious injuries among vulnerable road users is increasing [28]. In Europe, about one-third of road fatalities is caused by pedestrians and cyclists [29]. In Swedish, over 80% of serious injuries on urban roads are to pedestrians and cyclists [30]. Considering pedestrians and cyclists as an important part of road safety, enhancing and developing a safety culture that values serious injuries is what will provide safety for all vulnerable road users [31].

(3) Cluster 3 (Blue): Effect of speed on the severity

Keywords in cluster 3 (Blue) include: policy, speed, impact speed, severity, injury severity, collisions, vehicle, urban, road injury, design, etc.

In implementation research, policies play an important role. They are not only a starting point for implementation but also a reference point for future evaluations [32].

The development of transportation is reflected in time savings, which means the pursuit of faster speeds. However, the speed of vehicles is now considered a key factor in the likelihood and severity of accidents and has a great impact on road safety [33]. Speed dominates road casualties in terms of harm caused to organisms [34]. Lower speed limits can lead to lower average speeds, which makes fatal or serious injury accidents less likely [35]. The possibility of a road user being involved in a fatal accident increases as the speed of the vehicle exceeds the limit. In all highly motorized countries, speed is regulated by speed limits [36]. Establishing a speed control strategy is critical to the implementation of the zero-fatality vision.

(4) Cluster 4 (Yellow): Responsibility management for crashes

Keywords in cluster 4 (Yellow) include: crashes, safety, management, traffic fatalities, responsibility, accidents, ethics, culture, safe systems, organizations, etc.

From a traditional moral perspective, there seems to be a consensus that road users take full responsibility for the mistakes they make. However, without regulatory mechanisms to reduce the severity of crashes, the long-term plan of Vision Zero to eliminate traffic fatalities and serious injuries cannot be achieved [37]. Thus, Vision Zero proposed an innovative forward-looking concept of accident liability allocation, namely, that the road designer should be ultimately responsible for traffic accidents [38]. When road users fail to comply with the law, it is the responsibility of the road designer to take precautions to ensure the safety of road users. For instance, the installation of facilities, such as speed hills and speed tables in front of crosswalks can significantly reduce the fatalities of road users. It is worth noting that road safety cannot be achieved by a single organization, it requires the collaboration of multiple organizations to develop a systematic safety culture among the public.

### 3.3. Analysis of Countries and Regions

The analysis of countries and regions is to understand the research on the field by countries and regions through the institutions to which the different literature in a field belongs.

#### 3.3.1. The Number of Literature Results and Frequency of Citations

A total of 31 countries and regions are covered by the literature collected in this research. It should be noted that since the same literature may correspond to multiple countries and regions, the total amount of the literature here will be higher than the actual number of the literature.

As seen in Table 1, the United States is the most active country in the field of Vision Zero, with a total of 43 documents, accounting for 29.861% of the total. The literature in the United States has been cited 266 times, with an average frequency of 6.186 citations per paper. As the first country to adopt Vision Zero, Sweden published a total of 32 papers, accounting for 22.222% of the total. Sweden’s literature ranked first in total citations, with a total of 466 citations and an average frequency of 14.563 citations per paper. China, a rising important scientific force [39], has published a total of seven articles, accounting for 4.861% of the total, ranking 7th. Nevertheless, the literature in China has been cited a total of 205 times and has the highest average frequency of 29.286 citations per paper. Overall, the countries at the top of the literature output are either economically advanced or in a phase of rapid development.

#### 3.3.2. Time Dimension Distribution

**Figure 3:**
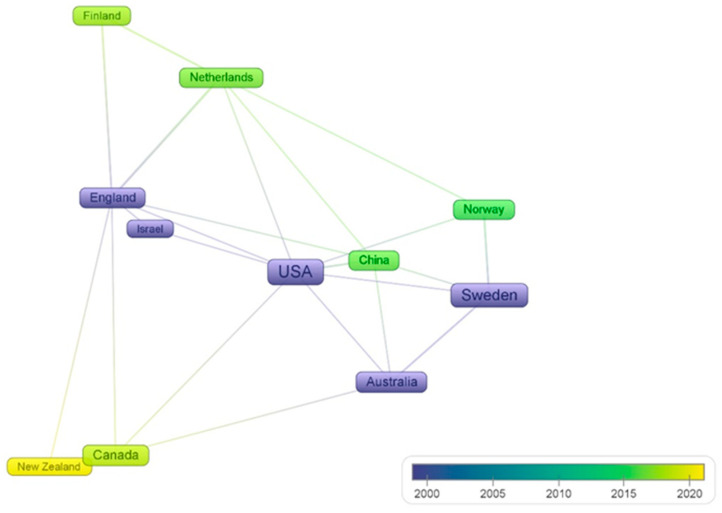
Distribution of major countries/regions in the time dimension.

Each country has its own unique context and varies in the time dimension of developing Vision Zero. Figure 3 illustrates the distribution on the timeline dimension for countries with no less than three articles in the literature.

Figure 3 clearly demonstrates that the publication of the literature in Vision Zero was dominated in the early years by Sweden, the United States, Australia, and England, followed by the rapid development of Vision Zero in China, Norway, and The Netherlands.

#### 3.3.3. Clustering of Countries and Regions

**Figure 4:**
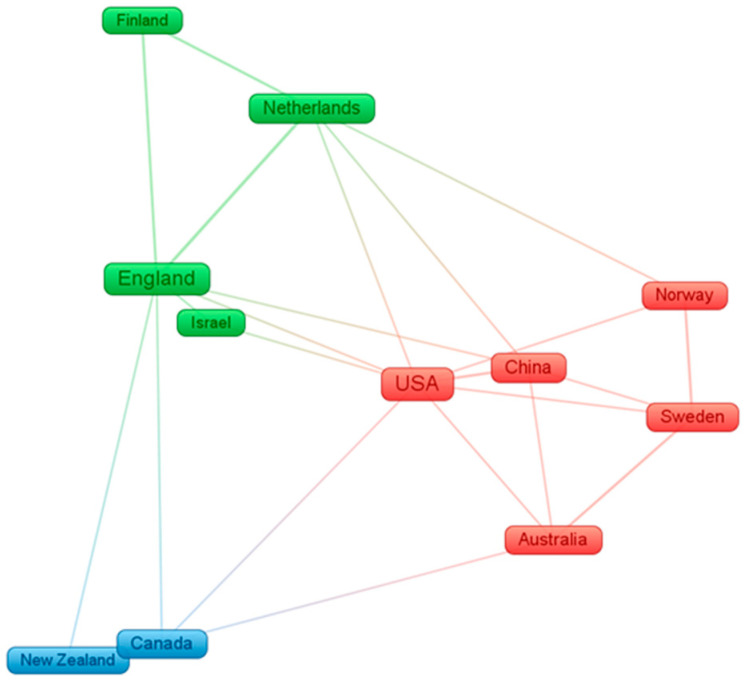
Cluster relationship network diagram of countries and regions.

The clustering of countries and regions provides a clear and unambiguous understanding of the relationship between individual countries and regions. Countries and regions clustered in the same cluster represent a high degree of correlation between them, facilitating subsequent discussion of individual countries and regions on Vision Zero area. There are 14 countries and regions with three or more literature results for all countries and regions, and Figure 4 shows the relationship network diagram for 12 of them that are associated.

According to Figure 4, it can be seen that countries and regions are clustered into a total of three clusters. Cluster 1 (red) covers five countries: Sweden, the United States, Australia, China, and Norway. England, Netherlands, Finland, and Israel make up cluster 2 (green). Canada and New Zealand form cluster 3 (blue). 

## 4. Status of Vision Zero

Now that Vision Zero has become the consensus for road safety development in several countries, it is very meaningful to understand the understanding and programs of Vision Zero in various countries. Combining Figure 3 and Table 1, it can be seen that the cluster consisting of Sweden, the United States, Australia, China, and Norway is more influential than the other clusters, both in terms of the number of literature results and number of citations. The current status of Vision Zero in these countries is presented below.

### 4.1. Sweden

As the first country to adopt Vision Zero, Sweden’s basic philosophy is that road users will make mistakes, so the road system needs a road tolerance to avoid all fatalities and serious injuries. Road tolerance is the design of a road system that can cause no more injuries than people can handle. Several Swedish roads have been redesigned based on the Vision Zero concept, and segregation is the most widely used principle.

- Additional lanes and separated vehicles.

When a vehicle is involved in a cross-median collision with an oncoming vehicle, it is one of the most serious types of collisions due to the high speed involved and the risk of a head-on collision [40]. Vision Zero uses the human body’s biological tolerance to external forces as a guidance mechanism to reduce the severity of collisions [41]. By increasing the number of lanes and medians to separate vehicles traveling in opposite directions, the probability of head-on collisions between vehicles traveling too fast or with a large weight difference is effectively reduced, significantly reducing the number of fatalities.

- Separating vulnerable road users, such as pedestrians and cyclists, from vehicles.

This concept of separating vulnerable road users and vehicles makes a positive contribution to the protection of vulnerable road users.

Through the above principles of separation of people and vehicles, the number of fatalities caused by road accidents in Sweden has continued to decrease during the implementation of Vision Zero, even though traffic volumes have been steadily increasing. Sweden is now the safest country in the world in terms of road safety. It is important to note, however, that in addition to fatalities, serious injuries continue to have a significant negative impact on public health and socioeconomics [42], and it has not yet been determined whether serious injuries are on the same downward trend as the number of fatalities [43].

### 4.2. The United States

The United States combines infrastructure projects with enforcement, education, and encouragement of cycling to achieve Vision Zero [44,45,46,47]. Different cities in the United States have different interpretations of Vision Zero, but three general points are the same [48].

- Vision Zero needs to be supported by policies.
- Vision Zero requires action plans and tracking indicators.
- Communication and cooperation between countries need to be strengthened.

As the largest city in the United States, road safety has always been a top priority for transportation development in New York City. New York City’s Vision Zero covers a wide range of areas including road design, traffic safety, culture, education, and traffic enforcement. Preventive law enforcement can be seen as an effective way to improve safety and reduce injuries [49]. Typical safety measures include redesigning road intersections, limiting vehicle speeds, and increasing penalties for drivers [50]. Moreover, the sustainable development of roads, especially chronic traffic safety, such as pedestrians and cyclists, is also a key concern of New York City. Since 2013, New York City has launched an autonomous bike-sharing program, which has significantly increased the accessibility of bicycles and the number of cyclists [51]. However, thousands of cycling injuries were reported in the same year. Some studies claim that helmets play an important role in cyclist crashes and that establishing helmet laws for cyclists could effectively advance the Vision Zero process [52]. In order to cope with the sustainable development of roads, New York City proposed the New York City pedestrian safety study and action plan in 2010 [53].

Nowadays, New York City has one of the lowest road fatality rates in the United States, and its road safety is significantly better than that of other comparable cities in the United States. Safer roads, safer vehicles, and richer measures are what New York City is looking for.

### 4.3. Australia

Australia has many similarities to the United States in terms of transportation, land use, and culture [54], yet does not adopt the same road safety policies. The Safe System is a framework specifically established for Australia, which combines Sweden’s Vision Zero and The Netherlands’ sustainable road safety philosophies [55]. This system has two basic key principles.

- A sustainable road system.

Humans have a limited tolerance for accidental injury. A safe road system should have a space to tolerate human errors, and any foreseeable accident injuries should be controlled within human’s bearing capacity.

- Safe road usage.

While Vision Zero holds the view that road designers should assume the ultimate safety of the road system, in road transport, Vision Zero can only be achieved progressively if road users comply with regulations. Australia has dealt strictly with a range of malpractices, such as speeding, drunk driving, and fatigued driving.

Safe roads, safe speeds, safe vehicles, and safe people are the four cornerstones of Australia’s national road safety strategy [56]. Safe roads are designed and maintained to prevent accidents. Safe speeds can reduce the harm to people caused by accidents by implementing speed limits on the road. Safe vehicles can simplify driving operations for drivers and provide protection for passengers. An unsafe attitude leads to increased risk acceptance [57]. Safe people encourage public safety through education and use law enforcement to restrict public behavior.

### 4.4. Norway

Norway adopted Vision Zero in 2001 on the basis of Sweden. Unlike Sweden, Norway focuses more on road users and more extensive road safety measures. In short, Vision Zero in Norway places greater emphasis on road safety in terms of road systems and traffic technology [58].

Norway’s official approach to implementing Vision Zero can be summarized as a science-based policy with enhanced monitoring of road system risks and increased attention to serious accidents and consequences [59]. More emphasis on the responsibility of road users and the lack of numerical standards, such as fatality rates, as in other countries, are features of Norway’s Vision Zero.

From the above descriptions, subject to cultural, economic, and social system and other factors, such as the current infrastructure, law enforcement levels vary from country to country, which also leads to different levels of road safety between different countries [60,61]. However, all countries are united in the notion of improving road safety and reducing public fatalities and injuries. Agenda 2030 aims to reduce road injuries by 50% between 2010 and 2020 and form safe cities by 2030 [43].

## 5. Road Safety in China

### 5.1. Analysis of Road Safety Indicators in China

**Figure 5:**
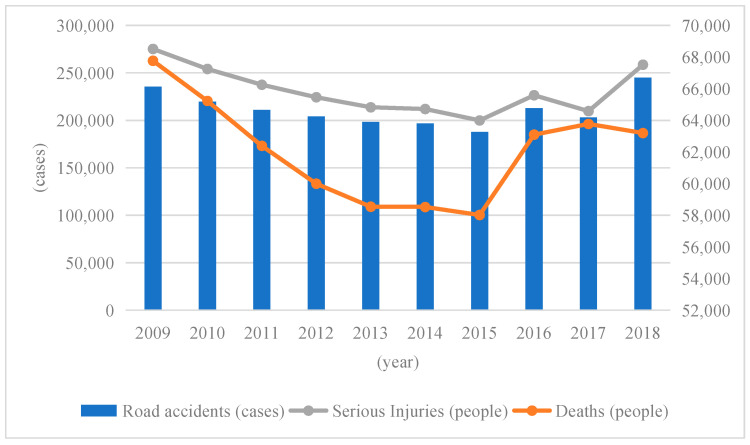
The number of road accidents and casualties in China from 2009 to 2018.

Road safety is determined by the number of accidents, fatalities, and serious injuries [62]. Figure 5 shows the number of road accidents, fatalities, and serious injuries in China from 2009 to 2018.

From Figure 5, it can be concluded that in 2018, the number of road accidents involving casualties in China was 244,937, with 63,194 fatalities and 258,532 serious injuries. Compared to 2017, the number of road accidents increased by 20.6%, while the number of fatalities decreased by 0.9%, and the number of injuries increased by 23.3%. Overall, the number of road accidents and serious injuries have been gradually decreasing in this decade, except for an increase in 2016 as well as in 2018. The number of fatalities, by contrast, declined again in 2018 after rising in 2016 and 2017.

### 5.2. Comparisons of Various Indicators between China and Other Countries

**Figure 6:**
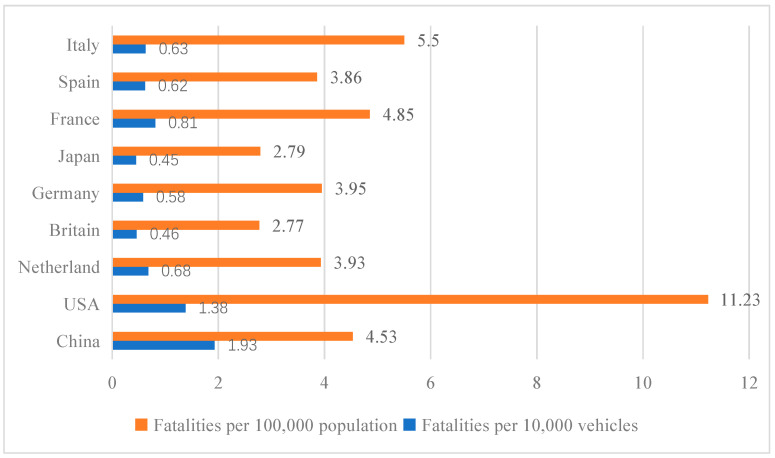
Comparisons of fatality rates between China and developed countries.

Due to the wide variation in both population density and the number of vehicles, it is not certain that the indicators in Figure 5 reflect the true situation when making international road safety comparisons. Considering the comparability, using the fatalities per 10,000 vehicles and the fatalities per 100,000 people provides a more objective description of the road safety level. Figure 6 displays the comparisons of the fatalities per 10,000 vehicles and the fatalities per 100,000 people between China and other countries in 2018.

As can be clearly seen in Figure 6, China had 1.93 fatalities per 10,000 vehicles in 2018, which is the highest among the countries listed, while the two countries with the lowest are Japan at 0.45 fatalities per 10,000 vehicles and the United Kingdom at 0.46 fatalities per 10,000 vehicles. Overall, all countries except China and the United States are below 1 fatality per 10,000 vehicles. In terms of the fatalities per 100,000 people, China has 4.53 fatalities per 100,000 people, which is in the middle of the range. The U.S. has the highest fatality rate at 11.23 per 100,000 people. In front of China are Italy with 5.5 fatalities per 100,000 people and France with 4.85 fatalities per 100,000 people.

### 5.3. Build China’s Strength in Transportation

Building China’s strength in transportation is a major strategic decision of China’s transportation, for which the Chinese Academy of Engineering has established a series of major consulting projects and formed “A Strategic Research on Traffic Safety Development” with Vision Zero as the goal. Guided by reducing accident mortality and improving system safety reliability, this study summarizes the development trend of traffic safety in China based on the analysis of the current situation of traffic safety, and proposes a traffic safety strategy of “a traffic safety vision, a development system of traffic safety, a support and guarantee system of traffic safety, and a set of tasks for improving traffic safety”, including:

- Achieve “Vision Zero”.
- Improve the comprehensive management and prevention system of traffic safety and the safety standards and technical system of transportation system.
- Build a comprehensive traffic safety emergency rescue system.
- Implement an innovation project for advanced transportation system safety technology and a key project to improve traffic safety.

### 5.4. Strategic Route toward Vision Zero

China is a country with a large road traffic network. As of the end of 2020, there were 456 million motorists in China, of which 418 million were car drivers. Additionally, the number of motor vehicles was 372 million, of which 281 million were cars [63]. With such a large number of road users and motor vehicles, the road safety situation in China is not optimistic. The complexity of road safety makes it impossible to consider all its aspects in any deterministic approach [64]. Therefore, it is important to learn from past accidents to reduce the likelihood of future accidents [65].

A successful strategic initiative should not only work in one area but benefit multiple areas [66]. A scientific and comprehensive strategic route of Vision Zero is necessary to effectively advance the process of Vision Zero. Realizing Vision Zero is the foundation for building a powerful country in transportation. The strategic route of road safety development in China is revealed in Table 2.

From Table 2, it can be concluded that China is currently at a stage of development in Vision Zero. The safety goal of China’s roads is to achieve a death rate of no more than 0.5 per 10,000 vehicles in 2030 and to basically achieve Vision Zero in 2045, that is, to achieve a death rate of no more than 0.3 per million vehicles in 10,000 vehicles. The road safety development system includes road safety comprehensive management, a prevention control system, and a road safety technology and standards system. The road safety development system will reach the international advanced level in 2030 and achieve the world’s leading position in 2045. In terms of advanced road system safety technology innovation projects, it is expected that information security and reliable control will be achieved in 2030, and network resilience and disaster recovery will be achieved in 2045. As for the road safety improvement project, it should be fully completed by 2030 [67].

### 5.5. Guidelines for Vision Zero in China

This chapter puts forward the following countermeasures and suggestions to promote the development of Vision Zero in China.

(1) Implement the main departments responsible for traffic safety supervision.

- Strengthen the comprehensive function of government supervision, innovate the supervision mode, and improve the supervision system.
- Under the leadership of the government, implement an integrated supervision mechanism and formulate goals, plans, and work measures in a unified manner to enhance the intensity and implementation of comprehensive traffic safety management.

(2) Improve safety of transportation infrastructure.

- Strengthen scientific and forward-looking transportation infrastructure planning and strengthen the safety design of transportation infrastructure.
- Improve the safety standard system of traffic infrastructure to ensure the quality and safety of traffic infrastructure construction.
- Strengthen the safety evaluation of traffic infrastructure, conduct regular survey and rectification of potential safety risks of traffic infrastructure, and establish a dynamic and intelligent feedback system of facility status.

(3) Strengthen the safety source management of vehicles.

- Strengthen the source management of vehicles, improve the safety and intelligence level of vehicles, and improve the technical status of vehicles.

(4) Vigorously carry out traffic safety publicity and education.

- Change the safety consciousness of traffic participants is the key and long-term countermeasure to improve the level of traffic safety.
- Continuously carry out effective, refined, and precise traffic safety publicity and education. Highlight publicity and education for key groups, innovate the concept of publicity and education, and enrich the form and content of publicity.
- Identify key groups to carry out multi-form, multi-way, targeted propaganda and education, strengthen drivers’ safety awareness, and develop good driving habits.

(5) Accelerate the traffic accident statistical standards in line with international standards.

- Formulate long-term accident statistics standards and norms, in line with international standards.
- Improve the direct reporting of accidents online to ensure timely, complete, and accurate statistical data.

## 6. Conclusions

Based on the methods of mapping knowledge domain and bibliometrics, this paper compares the research results in the field of Vision Zero in the Web of Science Core Collection database with the help of VOSviewer and comprehensively illustrates the development process, structural relationships, and status of Vision Zero. After displaying and comparing the road safety indicators of China and other countries, we provide a strategic route toward Vision Zero for China. The main features can be summarized as follows:

(1) The increasing number of papers in Vision Zero indicates the growing number of relevant studies conducted by the international academic community and the increasing emphasis on Vision Zero. The United States, Sweden, and Australia are the three countries with the highest number of literature results, and most of the literature produced in these three countries is between 2000–2010. Sweden, the United States, and China are the three countries with the highest frequency of literature citations, and most of the literature produced from China is between 2010–2020. More importantly, the five countries, the United States, Sweden, Australia, China, and Norway, were clustered into one cluster, representing a high correlation among these countries.

(2) By analyzing the keywords co-occurrence network diagram of Vision Zero, the hot research directions represented by these keywords can be divided into four categories, namely, “Vision Zero in road safety”, “vulnerable road users’ risks”, “effect of speed on severity”, and “responsibility management for crashes”. “Vision Zero in road safety” and “responsibility management for crashes” reflect the importance of the related concepts and policies of Vision Zero in road safety. In recent years, studies on “vulnerable road users’ risks” have emerged as society pays more and more attention to road users who are vulnerable to serious injuries and fatalities. Relevant research on “effect of speed on severity” shows the dominant role of speed in road accident casualties and the need for speed limits.

(3) Through the analysis of the number of road accidents, fatalities, and serious injuries in China, we found that all three indicators continued to decline between 2009 and 2015, but then all showed an upward trend after 2015, which indicates that the road safety situation in China is not stable. By comparing the fatalities per 10,000 vehicles and the fatalities per 100,000 people we can see that China’s death rate of 10,000 vehicles is 1.93, which is still a large gap with other countries. The death rate of 100,000 people is 4.53, which is in a medium level position. Combining these two indicators, we can conclude that China’s road safety still needs continuous improvement.

In this research, we focus on the research of visualization and analysis for global Vision Zero studies based on mapping knowledge domain (MKD) and bibliometric methods by the Web of Science (WOS) Core Collection database from 1997 to 2021. For future work, the studies in the research field of Vision Zero will be researched to further analyze the development of a certain research branch.

## Figures and Tables

**Table 1:** Top 10 productive countries, 2002–2021.

Rank	Countries/Territories	Quantity	Percentage	Citation	Frequency
1	the United States	43	29.861%	266	6.186
2	Sweden	32	22.222%	466	14.563
3	Australia	13	9.027%	193	14.846
4	Canada	12	8.333%	52	4.333
5	England	9	6.250%	73	8.111
6	Germany	9	6.250%	15	1.667
7	China	7	4.861%	205	29.286
8	Norway	7	4.861%	28	4
9	The Netherlands	7	4.861%	70	10
10	India	5	3.472%	16	3.2

**Table 2:** Strategic route of road safety.

Strategic Route of Road Safety Development						
Specific content	2020	2025	2030	2035	2040	2045
Safety goals	Fatalities per 10,000 vehicles ≤ 0.5			Fatalities per 10,000 vehicles ≤ 0.3		
Road safety development system	An internationally advanced safety system			A leading safety system in the world		
Advanced road system safety technology innovation project	Road system network safety			Advanced road system network resilience		
	Reliable vehicle control			Advanced road system disaster recovery		
Safety emergency rescue system	Complete safety and rescue emergency system					
	Comprehensively enhance the safety and reliability of the system					
Road safety improvement project	Support and safeguard system upgrading project					
	Cultural education enhancement project					
	Emergency rescue capacity improvement project					
	Prevention and control system upgrading project					
	Rural safety system construction project					

## Data Availability

Not applicable.

## References

[1] WHO (2018). Global Status Report on Road Safety 2018: Summary.

[2] Dekker S.W. (2014). The bureaucratization of safety. Saf. Sci..

[3] Elvik R. (1999). Can injury prevention efforts go too far?: Reflections on some possible implications of Vision Zero for road accident fatalities. Accid. Anal. Prev..

[4] Larsson P., Dekker S.W., Tingvall C. (2010). The need for a systems theory approach to road safety. Saf. Sci..

[5] Kim E., Muennig P., Rosen Z. (2017). Vision zero: A toolkit for road safety in the modern era. Inj. Epidemiol..

[6] Tremblay A., Badri A. (2018). Assessment of occupational health and safety performance evaluation tools: State of the art and challenges for small and medium-sized enterprises. Saf. Sci..

[7] Yang X., Wu C., He Y., Lu X., Chen T. (2022). A Dynamic Rollover Prediction Index of Heavy-Duty Vehicles with a Real-Time Parameter Estimation Algorithm Using NLMS Method. IEEE Trans. Veh. Technol..

[8] Moura R., Beer M., Patelli E., Lewis J., Knoll F. (2016). Learning from major accidents to improve system design. Saf. Sci..

[9] Khanzode V.V., Maiti J., Ray P.K. (2012). Occupational injury and accident research: A comprehensive review. Saf. Sci..

[10] Hultkrantz L., Lindberg G., Andersson C. (2006). The value of improved road safety. J. Risk Uncertain..

[11] Nord E., Richardson J., Street A., Kuhse H., Singer P. (1995). Who cares about cost? Does economic analysis impose or reflect social values?. Health Policy.

[12] Zhang W., Tsimhoni O., Sivak M., Flannagan M.J. (2010). Road safety in China: Analysis of current challenges. J. Saf. Res..

[13] He Y., Yang S., Chan C.-Y., Chen L., Wu C. (2020). Visualization Analysis of Intelligent Vehicles Research Field Based on Mapping Knowledge Domain. IEEE Trans. Intell. Transp. Syst..

[14] Yan X., Wu B., He Y., Zhong M., Zhu Y., Zhao X., Wang Q. (2019). A study of “Vision Zero” concept of transportation safety and its Impleementation strategies in China. J. Transp. Inf. Saf..

[15] Shiffrin R.M., Börner K. (2004). Mapping knowledge domains. Proc. Natl. Acad. Sci. USA.

[16] Zou X., Yue W.L., Le Vu H. (2018). Visualization and analysis of mapping knowledge domain of road safety studies. Accid. Anal. Prev..

[17] Van Eck N.J., Waltman L. (2010). Software survey: VOSviewer, a computer program for bibliometric mapping. Scientometrics.

[18] He Y., Feng Q., Yan L., Lu X.-Y. (2022). Visualization and analysis of mapping knowledge domain of heterogeneous traffic flow. Comput. Intell. Neurosci..

[19] Rosencrantz H., Edvardsson K., Hansson S.O. (2007). Vision Zero–is it irrational?. Transp. Res. Part A Policy Pract..

[20] Johansson R. (2009). Vision Zero–Implementing a policy for traffic safety. Saf. Sci..

[21] Strandroth J. (2015). Validation of a method to evaluate future impact of road safety interventions, a comparison between fatal passenger car crashes in Sweden 2000 and 2010. Accid. Anal. Prev..

[22] Zwetsloot G.I., Aaltonen M., Wybo J.-L., Saari J., Kines P., De Beeck R.O. (2013). The case for research into the zero accident vision. Saf. Sci..

[23] Kristianssen A.-C., Andersson R., Belin M.-Å., Nilsen P. (2018). Swedish Vision Zero policies for safety—A comparative policy content analysis. Saf. Sci..

[24] Sun Y., Huang Z., Zhao Z., Jiang Y., Ye Y., Yu T., Rao Y. (2014). Characteristics of 1226 alcohol-positive drivers involved in nonfatal traffic crashes in Shanghai, China. Traffic Inj. Prev..

[25] Zhang X., Zhao X., Du H., Ma J., Rong J. (2014). Effect of different breath alcohol concentrations on driving performance in horizontal curves. Accid. Anal. Prev..

[26] Evans L. (2004). Traffic Safety.

[27] Weijermars W., Bos N., Filtness A., Brown L., Bauer R., Dupont E., Martin J.L., Perez K., Thomas P. (2018). Burden of injury of serious road injuries in six EU countries. Accid. Anal. Prev..

[28] Värnild A., Larm P., Tillgren P. (2019). Incidence of seriously injured road users in a Swedish region, 2003–2014, from the perspective of a national road safety policy. BMC Public Health.

[29] Methorst R., Eenink R., Cardoso J., Machata K., Malasek J. (2016). Single unprotected road user crashes: Europe we have a problem!. Transp. Res. Procedia.

[30] Värnild A., Belin M.-Å., Tillgren P. (2016). 763 Vision Zero–Road Traffic Effects for Severely Injured in a Swedish County.

[31] Värnild A., Tillgren P., Larm P. (2020). What types of injuries did seriously injured pedestrians and cyclists receive in a Swedish urban region in the time period 2003–2017 when Vision Zero was implemented?. Public Health.

[32] Hill M., Hupe P. (2002). Implementing Public Policy: Governance in Theory and in Practice.

[33] Jurewicz C., Sobhani A., Woolley J., Dutschke J., Corben B. (2016). Exploration of vehicle impact speed–injury severity relationships for application in safer road design. Transp. Res. Procedia.

[34] Richter E.D., Berman T., Friedman L., Ben-David G. (2006). Speed, road injury, and public health. Annu. Rev. Public Health.

[35] Elvik R. (2013). A re-parameterisation of the Power Model of the relationship between the speed of traffic and the number of accidents and accident victims. Accid. Anal. Prev..

[36] Elvik R. (2010). A restatement of the case for speed limits. Transp. Policy.

[37] Cushing M., Hooshmand J., Pomares B., Hotz G. (2016). Vision Zero in the United States versus Sweden: Infrastructure improvement for cycling safety. Am. J. Public Health.

[38] Fahlquist J.N. (2006). Responsibility ascriptions and vision zero. Accid. Anal. Prev..

[39] Hollingsworth J.R., Müller K.H., Hollingsworth E.J. (2008). The end of the science superpowers. Nature.

[40] Chitturi M.V., Ooms A.W., Bill A.R., Noyce D.A. (2011). Injury outcomes and costs for cross-median and median barrier crashes. J. Saf. Res..

[41] Johnston I. (2010). Beyond “best practice” road safety thinking and systems management—A case for culture change research. Saf. Sci..

[42] Bambach M.R., Mitchell R.J. (2015). Estimating the human recovery costs of seriously injured road crash casualties. Accid. Anal. Prev..

[43] Värnild A., Tillgren P., Larm P. (2020). Factors related to the increasing number of seriously injured cyclists and pedestrians in a Swedish urban region 2003–2017. J. Public Health.

[44] Reynolds S., Gale N. (2016). How to think big. Int. J. Traffic Saf. Innov..

[45] Gonzalez S. (2016). The political will to save lives. Vis. Zero Cities Int. J. Traffic Saf. Innov..

[46] Territo C. (2016). The power of automated enforcement. Vis. Zero Cities Int. J. Traffic Saf. Innov..

[47] Shahum L. (2016). Vision Zero by the people. Vis. Zero Cities Int. J. Traffic Saf. Innov..

[48] Naumann R.B., Heiny S., Evenson K.R., LaJeunesse S., Cooper J.F., Doggett S., Marshall S.W. (2019). Organizational networks in road safety: Case studies of US Vision Zero cities. Traffic Inj. Prev..

[49] Behera R.K., Hassan M.I. (2019). Regulatory interventions and industrial accidents: A case from India for ‘Vision Zero’goals. Saf. Sci..

[50] Mendoza A.E., Wybourn C.A., Mendoza M.A., Cruz M.J., Juillard C.J., Dicker R.A. (2017). The worldwide approach to Vision Zero: Implementing road safety strategies to eliminate traffic-related fatalities. Curr. Trauma Rep..

[51] Basch C.H., Ethan D., Rajan S., Samayoa-Kozlowsky S., Basch C.E. (2014). Helmet use among users of the Citi Bike bicycle-sharing program: A pilot study in New York City. J. Community Health.

[52] Sethi M., Heidenberg J., Wall S.P., Ayoung-Chee P., Slaughter D., Levine D.A., Jacko S., Wilson C., Marshall G., Pachter H.L. (2015). Bicycle helmets are highly protective against traumatic brain injury within a dense urban setting. Injury.

[53] Viola R., Roe M., Shin H.-S. (2010). New York City Pedestrian Safety Study & Action Plan.

[54] Marshall W.E. (2018). Understanding international road safety disparities: Why is Australia so much safer than the United States?. Accid. Anal. Prev..

[55] Corben B.F., Logan D.B., Fanciulli L., Farley R., Cameron I. (2010). Strengthening road safety strategy development ‘Towards Zero’ 2008–2020–Western Australia’s experience scientific research on road safety management SWOV workshop 16 and 17 November 2009. Saf. Sci..

[56] Hughes B., Anund A., Falkmer T. (2015). System theory and safety models in Swedish, UK, Dutch and Australian road safety strategies. Accid. Anal. Prev..

[57] He Y., Sun C., Huang H., Jiang L., Ma M., Wang P., Wu C. (2021). Safety of micro-mobility: Riders’ psychological factors and risky behaviors of cargo TTWs in China. Transp. Res. Part F Traffic Psychol. Behav..

[58] Valen A., Bogstrand S.T., Vindenes V., Frost J., Larsson M., Holtan A., Gjerde H. (2019). Fatally injured drivers in Norway 2005–2015—Trends in substance use and crash characteristics. Traffic Inj. Prev..

[59] Elvebakk B., Steiro T. (2009). First principles, second hand: Perceptions and interpretations of vision zero in Norway. Saf. Sci..

[60] Gaygısız E. (2010). Cultural values and governance quality as correlates of road traffic fatalities: A nation level analysis. Accid. Anal. Prev..

[61] Castillo-Manzano J.I., Castro-Nuño M., Pedregal D.J. (2011). Can fear of going to jail reduce the number of road fatalities? The Spanish experience. J. Saf. Res..

[62] Eksler V. (2010). Measuring and understanding road safety performance at local territorial level. Saf. Sci..

[63] Burea T.A. 33.28 Million Newly Registered Motor Vehicles Nationwide in 2020 New Energy Vehicles Reached 4.92 Million. https://app.mps.gov.cn/gdnps/pc/content.jsp?id=7647257.

[64] Salmon P.M., Lenné M.G. (2014). Miles away or just around the corner? Systems thinking in road safety research and practice. Accid. Anal. Prev..

[65] Ruj B., Chatterjee P.K. (2012). Toxic release of chlorine and off-site emergency scenario—A case study. J. Loss Prev. Process Ind..

[66] McIlroy R.C., Plant K., Hoque M., Wu J., Kokwaro G., Nam V., Stanton N. (2019). Who is responsible for global road safety? A cross-cultural comparison of Actor Maps. Accid. Anal. Prev..

[67] Fu Z., Sun Y., Weng M., He H. (2019). Strategic Research on Transportation Power.

